# The impact of immediate versus delayed mini-screw placement on alveolar bone preservation and bone density following tooth extraction: evidence from a canine model

**DOI:** 10.1186/s12903-023-03703-7

**Published:** 2023-12-06

**Authors:** Arezoo Jahanbin, Neda Eslami, Hamideh Salari Sedigh, Narges Ghazi, Seyed Hossein Hosseini Zarch, Melika Hoseinzadeh, Sedigheh Moayedi

**Affiliations:** 1 Department of Orthodontics, School of Dentistry, Mashhad University of Medical Sciences, Mashhad, Iran; 2 Department of Clinical Sciences, Faculty of Veterinary Medicine, Ferdowsi University of Mashhad, Mashhad, Iran; 3 Department of Oral and Maxillofacial Pathology, School of Dentistry, Mashhad University of Medical Sciences, Mashhad, Iran; 4 Department of Oral and Maxillofacial Radiology, School of Dentistry, Mashhad University of Medical Sciences, Mashhad, Iran; 5 Dentist, Research Assistant, Dental Research Center, Mashhad Dental School, Mashhad University of Medical Sciences, Mashhad, Iran; 6 Orthodontics Research Center, Department of Orthodontics, School of Dentistry, Shiraz University of Medical Sciences, Shiraz, Iran

**Keywords:** Alveolar bone, Atrophy, Mini-screw, Density, Radiology, Histology

## Abstract

The aim of this study was to evaluate the impact of mini-screw placement on the alveolar ridge using a split-mouth design. Twelve beagles underwent bilateral extraction of their lateral teeth. In the immediate group, a mini-screw was unilaterally placed approximately 3–4 mm below the alveolar crest of the extraction site on the experimental side. The delayed group received mini-screws six weeks after tooth extraction. On average, the dogs were sacrificed after 11 weeks, and the maxillary bones were excised and scanned using cone-beam computed tomography (CBCT). Histopathological examinations were conducted to assess inflammation and bone formation scores. The results showed that in the immediate group, bone height was significantly greater on the intervention side compared to the control side (*p* < 0.05), whereas there was no significant difference in the delayed group. In both groups, there was a significant increase in bone density around the mini-screws compared to the control sides (*p* < 0.05). Mini-screw insertion led to a significant enhancement of bone growth in both groups (*p* < 0.05), with no notable differences between the two groups. The mini-screws did not have any impact on bone inflammation or width. Overall, both immediate and delayed mini-screw placement in the extraction socket positively influenced bone dimensions, density, and histological properties. However, immediate insertion was more effective than delayed placement in preserving vertical bone height, despite delayed insertion resulting in higher bone density.

## Introduction

Tooth loss often leads to significant resorption of the alveolar ridge, resulting in a substantial reduction of bone height and width, typically ranging from 40 to 60% [1]. Preserving the alveolar bone is crucial to minimize the need for complex reconstructive surgeries [2]. However, in cases such as congenitally missing teeth or cleft patients, immediate dental implant placement is often delayed due to ongoing skeletal growth or deteriorating bone grafts, making it challenging to replace missing teeth, particularly the lateral incisors [3]. Therefore, ridge preservation after tooth extraction is essential for successful dental implant placement, especially in cases where immediate placement is not possible due to remaining skeletal growth, cleft patients, the patient’s systemic condition, or the patient’s financial situation.

Various methods and materials, including bone products, biologically active substances, and immediate dental implants, have been proposed for alveolar bone preservation and reconstruction [4–7]. However, these procedures have drawbacks, such as high costs, inadequate blood supply to grafted areas, unpredictable graft resorption, and harvesting restrictions [8]. Additionally, the use of platelet concentrates, such as platelet-rich fibrin (PRF), is currently limited to clinical trials [9, 10]. Therefore, there is a need to explore simple, cost-effective, and efficient techniques to conserve alveolar bone after tooth extraction.

Mini-screws, commonly employed as temporary anchorage devices in orthodontics, have shown a success rate of 85.21% [11]. Bone density increases around mini-screws that are loaded [12–14]. Studies have demonstrated comparable bone-implant contact between loaded and unloaded mini-screws [2, 15]. However, there is limited research on the effect of unloaded mini-screws specifically on the alveolar ridge. Although studies have shown that unloaded transcortical mini-screws can maintain the alveolar ridge in beagle dogs after mandibular tooth extraction [2], the distinct blood supply characteristics of the maxillary bone and the requirements of the esthetic zone necessitate further investigation. Some case reports have documented successful use of mini-screws to preserve bone around missing or lost maxillary incisors in growing patients [16–18]. However, larger studies with comprehensive analyses, particularly in the aesthetic zone, are lacking.

Therefore, the aim of this study was to evaluate the immediate or delayed effect of mini-screw insertion on ridge height, width, density, and histologic features in a canine model following extraction of the maxillary lateral incisors. By providing valuable insights into the efficacy and feasibility of mini-screw insertion as a ridge preservation technique, our findings may have significant implications for future clinical applications.

## Methods and materials

The protocol of the current animal study was reviewed and approved by the Research and Ethics Committee of Mashhad University of Medical Sciences (IR.MUMS.DENTISTRY.REC.1400.041). The present study is reported in accordance with ARRIVE guidelines.

### Sample size calculation

According to our observations regarding the density at zero surface of the intervention sides of the immediate (1319.0 **±** 116.2 Hu) and delayed groups (1504.2 **±** 147.5 Hu) performed a power analysis using the G*Power software. By using the formula designed for comparing two independent samples, and taking into account an alpha of 0.05 and a beta of 0.20, we established that eight dogs in each group would be ideal. Sixteen two-year-old male beagles with a mean weight of 8 ± 1 kg were bought from Mashhad’s municipality for this split-mouth animal study. Unfortunately, due to mini-screw instability, four dogs had to be sacrificed prior to the anticipated study duration. Consequently, each group was left with six dogs remaining for the study. However, four dogs were sacrificed before the considered study time due to mini-screw instability; therefore, six dogs were remained in each group.

### Study design and surgical procedure

The animals had the opportunity for adaptation to the housing and diet for 2 weeks prior to the operation. In this period, vaccination and deworming treatments were carried out. All the dogs received polyvalent and rabies vaccines (Biocan, Bioveta, Czech) as well as antiparasitic treatment (Ivermectin SC, Alfasan and Topkim tablets, Turkey). During the whole experiment, all animals were kept individually in cages, fed once a day with soft diet for dogs and monitored for general appearance, activity, exertion and feeding.

During surgery, anesthesia was induced with an intravenous administration of ketamine hydrochloride (10 mg/kg) and diazepam (0.5 mg/kg), following pre-medication with intramuscular acepromazine (0.01 mg/kg). Hemostasis was achieved using 2% lidocaine and 1:50.000 epinephrine (0.5 ml), after which the maxillary lateral incisors (teeth 102 and 202) were extracted bilaterally. Absorbable 4 − 0 sutures were used to close the extraction sites.

**Fig. 1:**
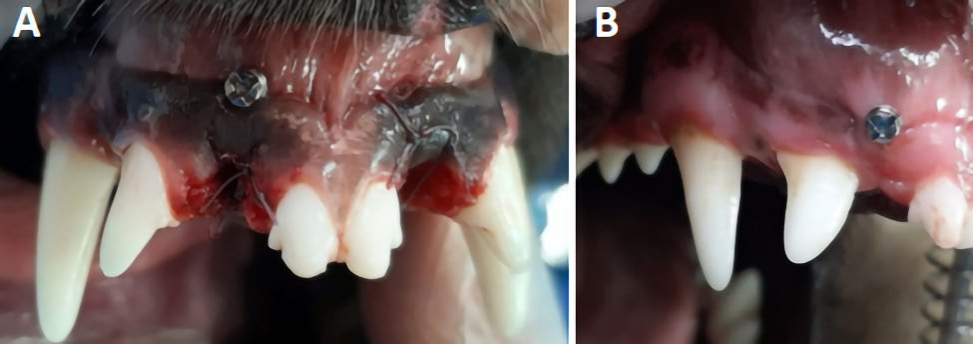
(**A**) Screw insertion on the intervention side in the immediate group, and (**B**) the delayed group (original image)

Post-extraction, each dog in the immediate group (N = 6) had a titanium mini-screw inserted apical to the crestal bone. The mini-screws, sourced from Jeil Medical Corporation (Guro-gu, Seoul, Republic of Korea), were 1.5 mm in diameter and 6.5 mm in length and placed 3–4 mm apical to the crestal bone (Fig. 1-(A)). One milligram of cefazolin was delivered intravenously one hour before and immediately after surgery.

The delayed group (N = 6) had the mini-screws inserted six weeks post tooth extraction (Fig. 1-(B)). Throughout the trial, screw stability was assessed daily. All animals maintained their weight. The interventions (tooth extraction and mini-screw insertion) were carried out by the same operator.

On average, 11 weeks post mini-screw insertion, the animals were euthanized via a sodium thiopental overdose. Their maxillary bones were extracted for CBCT and microscopic analysis. The mini-screws were removed to mitigate artifacts.

### CBCT examination

**Fig. 2:**
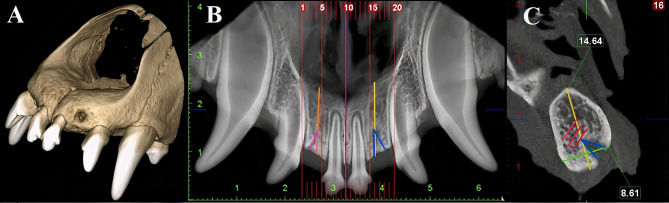
Measuring the bone height and density and the ridge width. (**A**) 3D view of the dog’s jaw. (**B**) On the panoramic-like view of CBCT, Sect. 16 was determined as the screw entrance section (blue triangle), and on the control side, the corresponding section (Sect. 4) was selected (purple triangle). (**C**) The same section was identified in the sagittal view. The height (yellow line) and width (green line) were determined in Sect. 16. In these samples, the bone height and ridge width were 14.64 and 8.61 mm, respectively. Bone density at 0, 1 mm, and 2 mm levels perpendicular to the apical region of the screw entrance were determined (red lines) (original image). The same measurements were conducted on the control side in Sect. 4 (original image)

Radiographic images of each maxilla were captured using a CBCT device (Planmeca Promax G7, Helsinki, Finland) with a 75 μm voxel size (Fig. 2-A). Bone height and width measurements were taken from the panoramic-like and sagittal view cross-sections at 1 mm intervals, respectively. The bone height was measured from the nasal cavity floor to the alveolar bone crest at the section corresponding to the screw entry point (Fig. 2- (B, C)). The bone width was measured by calculating the distance between the buccal and lingual cortical bones at the same section (Fig. 2- (C)). On the control side, measurements were made relative at the same levels to the midline.

On the axial view, bone density was measured at three levels from the screw entry point: (zero level) at the apical point of screw insertion, (1 mm level) 1 mm apical to zero level, and (2 mm level) 2 mm apical to zero level (Fig. 2-C). These measurements were made in a circle with a 10 mm diameter drawn at each level. A 200-micron slice thickness was used to calculate the mean bone density values, excluding the adjacent teeth’s lamina dura and cortical bone. On the control side, bone density was measured at the same levels in relation to the intermaxillary suture.

### Histopathological examination and histomorphometric measurements

Post CBCT examination, the specimens were preserved in formalin, decalcified with ethylenediaminetetraacetic acid (EDTA), processed, and encased in paraffin [19]. Based on the criteria from Ali-erdam et al. [20], osteoid formation was categorized. The osteoid tissue presence was classified as mild (score 1: 1–30%), moderate (score 2: 30–60%), and severe (score 3: more than 60%).

To obtain histomorphometric measurements, a single calibrated examiner, masked to the treatment codes examined the extension of osteoid covered surfaces in each specimen photographed with a camera (TrueChrome Metrics, Tucsen, China) coupled to a light microscope (LABOMED, Labo America Inc., California, USA) with a 40× objective using ImageJ software.

### Statistical analysis

Statistical analysis was performed using SPSS software, version 21.0 (IBM, New York, NY, USA). Mean values and standard deviations were determined for all parameters. The independent t-test or the Mann-Whitney test was used to assess the quantitative variables collected from CBCT scans. The Chi-square and Fisher’s exact tests were used to compare the presence of inflammation and bone formation scores between groups. The paired t-tests and Wilcoxon tests were used to compare the intervention and control sides. The significance level was set at 0.05.

## Results

In the delayed group, two mini-screws became loose on day 61. Meanwhile, two mini-screws in the immediate group loosened on days 63 and 66. As a response to these findings, the dogs were euthanized 48 h following these observations and they were not included in the measurement. Throughout the entirety of the study, the other mini-screws remained stable. The duration for which mini-screws remained in the dogs’ jaws averaged 82 days for the immediate group and 79 days for the delayed group, with no statistically significant difference between these time periods (*p* = 0.847).

### Bone density

Regarding bone density, CBCT measurements indicated a higher average bone density at the zero level on the experimental side (1411.6 ± 175.9 Hu) compared to the control side (1276.7 ± 159.3 Hu) across all samples (*p* < 0.001) (Table 1). The difference in bone density between the experimental and control sides was significantly larger in the delayed group (183.3 ± 86.3 Hu) than the immediate group (86.5 ± 50.0 Hu) (*p* = 0.025). The experimental side of the delayed group also exhibited significantly greater bone density (1504.2 ± 147.5 Hu) than the immediate group (1319.0 ± 116.2 Hu) (*p* = 0.036).

**Table 1:** Inter-group and intra-group differences of density (Hu) in zero surface in the immediate and delayed groups

Sides	Group			*P*-value
	Delayed	Immediate	Total	
Control	1320.8 **±** 193.4	1232.5 **±** 161.3	1276.7 **±** 175.9	0.410
Intervention	1504.2 **±** 147.5	1319.0 **±** 116.2	1411.6 **±** 159.3	0.036 ^a)^
Difference between the Intervention and Control sides	183.3 **±** 86.3	86.5 **±** 50.5	134.9 **±** 84.3	0.025 ^b)^
*P*-value	0.003 ^c)^	0.009 ^c)^	< 0.001 ^c)^	

Values are presented as mean ± standard deviation of the bone density (Hu)

^a)^ Statistically significant difference between delayed and immediate groups using the independent *t*-test (*P* < 0.05)

^b)^ Statistically significant difference between delayed and immediate groups using the Mann–Whitney U-test (*P* < 0.05)

^c)^Statistically significant difference between control and intervention side using the paired *t*-test (*P* < 0.05)

At the 1 mm level, the experimental side of all samples (1289.9 ± 138.5 Hu) displayed a higher average bone density than the control side (1245.7 ± 131.5 Hu) (*p* = 0.001) (Table 2). In the delayed group, the experimental side’s density was significantly greater than the control side’s (*p* = 0.027). However, in the immediate group, bone density at the 1 mm level did not differ significantly from the control side. The delayed group tended to have a higher screw-side density than the immediate group, but this difference was not statistically significant.

**Table 2:** Inter-group and intra-group differences of bone density (Hu) 1 mm apical to mini-screws in the immediate and delayed groups

Sides	Group			*P*-value
	Delayed	Immediate	Total	
Control	1295.8 **±** 99.2	1199.5 **±** 150.5	1247.7 **±** 131.5	0.631
Intervention	1355.3 **±** 99.8	1224.5 **±** 148.3	1289.9 **±** 138.5	0.200
Difference between the Intervention and Control sides	59.5 **±** 10.0	25.0 **±** 37.3	42.2 **±** 31.6	0.053
*P*-value	0.027 ^a)^	0.161	0.001 ^a)^	

Values are presented as mean **±** standard deviation of the bone density (Hu)

^a)^ Statistically significant difference between control and intervention side using the paired *t*-test (*P* < 0.05)

Contrary to the observed trends in bone density at other levels, no significant difference was found in bone density 2 mm apical to the mini-screw between the experimental and control sides in any of the samples (Table 3).

**Table 3:** Inter-group and intra-group differences of bone density (Hu) 2 mm apical to mini-screws in the immediate and delayed groups

Sides	Group			*P*-value
	Delayed	Immediate	Total	
Control	1272.7 **±** 107.6	1177.8 **±** 120.7	1225.2 **±** 119.8	0.181
Intervention	1306.5 **±** 96.7	1182.5 **±** 104.1	1244.5 **±** 115.7	0.058
Difference between the Intervention and Control sides	33.8 **±** 34.8	4.7 **±** 43.4	19.2 **±** 40.5	0.228
*P*-value	0.063	0.803	0.128	
Values are presented as mean **±** standard deviation of the bone density (Hu) in zero surface				

### Bone height

Regarding bone height, the insertion of mini-screws resulted in a significantly higher bone height (0.3 ± 0.3 mm) on the experimental side across all samples (*p* = 0.014) (Table 4). In the immediate group, the experimental side bone height (12.1 ± 1.4 mm) was significantly higher than the control side (11.7 ± 1.3 mm) (*p* = 0.02). However, in the delayed group, no significant difference was identified. The immediate group demonstrated a significantly greater difference in bone height between the experimental and control sides (0.4 ± 0.3 mm) than the delayed group (0.1 ± 0.1 mm) (*p* = 0.029).

**Table 4:** Inter-group and intra-group differences of the alveolar bone height (mm) in immediate and delayed groups

Sides	Group			*P*-value
	Delayed	Immediate	Total	
Control	11 **±** 2.6	11.7 **±** 1.3	11.3 **±** 2.0	0.567
Intervention	11.1 **±** 2.6	12.1 **±** 1.4	11.6 **±** 2.1	0.394
Difference between the Intervention and Control sides	0.1 **±** 0.1	0.4 **±** 0.3	0.3 **±** 0.3	0.029 ^a)^
*P*-value	0.160	0.023 ^b)^	0.014 ^b)^	

Values are presented as mean **±** standard deviation of the alveolar bone height (mm)

^a)^ Statistically significant difference between delayed and immediate groups using the independent *t*-test (*P* < 0.05)

^b)^ Statistically significant difference between control and intervention side using the paired *t*-test (*P* < 0.05)

### Bone width

As for bone width, the insertion of mini-screws did not significantly affect ridge width. Both the experimental and control sides across all samples exhibited comparable ridge widths (Table 5), with a difference of 0.2 ± 0.3 mm observed in both groups.

**Table 5:** Inter-group and intra-group differences of alveolar bone width (mm) in the immediate and delayed groups

Sides	Group			*P*-value
	Delayed	Immediate	Total	
Control	5.2 **±** 0.9	6.1 **±** 1.3	5.6 **±** 1.1	0.183
Intervention	5.3 **±** 0.9	6.3 **±** 1.6	5.8 **±** 1.3	0.243
Difference between the Intervention and Control sides	0.1 **±** 0.3	0.2 **±** 0.3	0.2 **±** 0.3	0.899
*P*-value	0.166	0.201	0.053	

Values are presented as mean **±** standard deviation of the alveolar bone width (mm)

### Inflammation levels

**Fig. 3:**
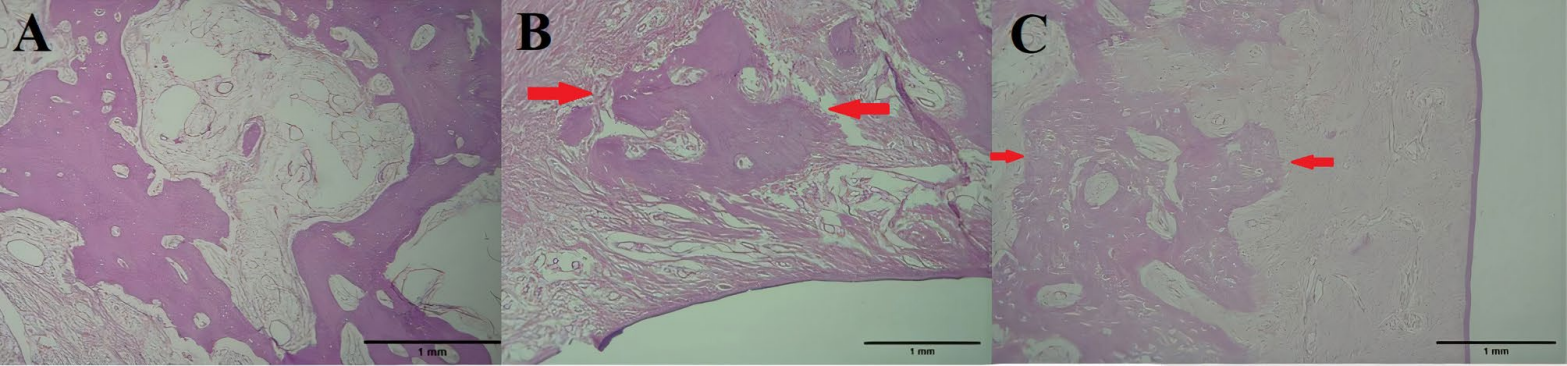
Histopathological samples with 40x magnification, H&E staining. (**A)** Control side sample with no new bone formation and presence of inflammation. (**B**) Intervention side of a sample in the immediate group with score 2 new bone formation. (**C**) The intervention side of the sample in the delayed group with score of 3 bone formation with no inflammation. The red arrow represents new bone formed in the samples according to histopathological criteria (original image)

In terms of inflammation levels, the histopathological evaluation revealed no significant differences between the experimental and control sides in either group. Signs of inflammation were observed in one sample from the control side of the delayed group (Fig. 3- A) and one from the experimental side of each group (Fig. 3- B, C).

### Bone formation

Regarding bone formation scores, the placement of the screws led to new bone formation in all samples (Fig. 3- B, C). On the experimental side of all samples, the average score for osteoid formation (2.6 ± 0.8) was significantly higher than on the control side (0.1 ± 0.3) (*p* = 0.002) (Table 6). In the delayed group, the osteoid formation score on the experimental side (2.5 ± 0.8) was also significantly higher than the control side (0.2 ± 0.4) (*p* = 0.034). In the immediate group, the score on the experimental side was 2.7 ± 0.5, whereas no bone formation was observed on the control side (*p* = 0.023). However, the bone formation scores did not significantly differ between the immediate and delayed groups.

**Table 6:** Inter-group and intra-group differences of bone scores in the immediate and delayed groups

Sides	Group			*P*-value
	Delayed	Immediate	Total	
Control	0.2 **±** 0.4	0.0 **±** 0.0	0.1 **±** 0.3	0.317
Intervention	2.5 **±** 0.8	2.7 **±** 0.5	2.6 **±** 0.7	0.847
Difference between the Intervention and Control sides	2.3 **±** 1.2	2.7 **±** 0.5	2.5 **±** 0.9	0.847
*P*-value	0.034 ^a)^	0.023 ^a)^	0.002 ^a)^	

Values are presented as mean **±** standard deviation of scores

^a)^ Statistically significant difference between control and intervention side using the Wilcoxon signed-rank test (*P* < 0.05)

The histomorphometric analysis revealed that in the delayed group the mean osteoid extension in the intervention side was significantly higher than the control side (6.086 ± 0.803 versus 0.182 ± 0.125 mm^2^; *p* = 0.033). Similarly, in the immediate group, new bone formation was significantly higher on the intervention side (4.27 ± 0.161 versus 0.037 ± 0.008 mm^2^; *p* < 0.001). However, the finding of the independent sample t-test revealed that the difference between the intervention sides of the groups was not significantly different (*p* = 0.277).

## Discussion

Following tooth loss, congenital tooth agenesis, or bone grafting, physiological or pathological bone resorption occurs [1, 21]. After orthodontic treatment, implant insertion is an option for individuals with congenital maxillary lateral incisor agenesis. However, this treatment may not be immediately feasible owing to continuous growth or economic concerns [1, 2]. Multiple studies have demonstrated that the alveolar ridge’s width and height decrease significantly in these patients [22, 23].

To the best of our knowledge, this was the first research to investigate and evaluate the impact of immediate or delayed mini-screw insertion on the extraction socket bone height, width, density, and histopathological characteristics in an animal model. Mini-screws are usually inserted in areas with adequate bone density and minimal turnover for stability reasons [24]. Similar to Melsen et al. [2], in our study, mini-screws were inserted with no external loading at the extraction socket, where dynamic healing occurs.

Inserting the mini-screw immediately after tooth extraction significantly preserved the alveolar bone height compared to the control side (on average, 12.1 mm versus 11.7 mm). In contrast, no significant differences in ridge height between the intervention and control sides were detected in the delayed group. Despite the findings of our delayed study group, Melsen et al. [2] demonstrated that the alveolar bone height on the intervention side was considerably higher than the control side at week 12 when mini-screws were placed six weeks following tooth extraction. Our larger sample size and differences in the characteristics of the mandibular and maxillary bones may account for the different outcomes. Generally, the maxilla tends to have a better blood supply and may heal faster than the mandible. According to the result of a study by Zhang et al. [25] on beagle dogs, mini-screws inserted in maxilla showed better stability and trabecular bone volume density compared to the one inserted in the mandible in 7 weeks. Therefore, healing may happen in shorter period of time and led to insignificant difference between the intervention and control sides.

In our study, the observation time following screw placement was 12 weeks, according to Melsen et al. [2]. Evidence suggests that bone resorption decreases after 12 weeks. For example, in one study, the augmented sockets resorbed the most between 4 and 6 weeks after tooth extraction, whereas the non-augmented sockets continued to resorb for up to 12 weeks. At week 12, the resorption rate in augmented and non-augmented tooth sockets became comparable [26]. Another research discovered that by week six after extraction, the alveolar bone height on the buccal, middle, and lingual sides had fallen to 53%, 77%, and 85.5%, respectively. By week 12, the remaining bone height on the buccal, middle, and lingual sides was about 49%, 86.5%, and 84%, respectively. The most substantial reduction in bone ridge height was noted on the buccal side during the first six weeks; however, there were few changes between the sixth and twelfth weeks [27]. Consequently, it seems that the first six weeks after extraction are the most susceptible to dimension changes, and early management is required during this period. Our study suggests that the immediate placement of mini-screws may result in higher preservation of bone height than the delayed placement, particularly for the buccal plate of the lateral incisors, which are located in the aesthetic zone. Intriguingly, the difference between the intervention and control groups was much more noticeable in the immediate group than in the delayed group (on average, 0.4 mm in the immediate group versus 0.1 mm in the delayed group).

The ridge width in either of the groups was unaffected by the mini-screws. The short follow-up time in our study may help explain this result. Long-term insertion of mini-screws may alter the alveolar ridge’s width. In a two-year follow-up, Choi et al. [28] reported that mini-screws placed 2–3 mm apical to the alveolar bone margin maintained bone width in a child with mandibular incisor agenesis. However, more research is required to assess mini-screws’ impact on bone width.

In the delayed group, bone density at zero level and 1 mm apical to the mini-screw was higher than in the control group. Consistent with these observations, Melsen et al. [2] also showed an increase in ossification around mini-screws inserted six weeks after tooth extraction. According to their research, the bone volume around the mini-screws and the virtual dental implant on the control side was considerably different after 12 weeks [2]. In our investigation, bone density was assessed at three levels; however, there was no difference between the bone density at 2 mm apical to the mini-screws and the control side in either group. In addition, all samples (immediate and delayed) showed an increase in bone density at the level of 1 mm apical to the mini-screws entry point. Therefore, it appears that mini-screws have a positive effect on their surrounding bone density.

In contrast to our study, Melsen et al. [2] evaluated bone density using micro-CT. Although micro-CT and multislice-CT are the gold standards for evaluating morphology, microstructure, and bone density, CBCT is more accessible, has a lower cost and radiation dose, and is utilized more frequently in dentistry. In addition, CBCT has been validated as a tool for assessing dental implants in comparison to multislice CT [29–31].

In the immediate group, bone density around the mini-screws was much higher than in the control group. In contrast, there was no difference in bone density 1 and 2 mm apical to the mini-screw between the control and experimental sides. In the delayed group, there was a significant difference in bone density at zero level and 1 mm apical to the mini-screw between the experimental and control sides. Choi et al. discovered that the bone density of extraction sockets in beagle dogs increased during the first four weeks after tooth extraction and stabilized by the sixth week. In the extraction socket, bone deposition began one week after extraction. In the fifth week, hard tissue was deposited at the entrance of the socket [32]. We postulate that the insertion of mini-screws in relatively restored tooth sockets may cause a synergistic release of inflammatory mediators that leads to better results in the delayed group compared to the immediate group in terms of bone density.

According to the histopathological findings and histomorphometric analysis of our study, inserting unloaded screws resulted in significantly greater bone formation scores than the control sides, regardless of whether the mini-screws were inserted immediately or delayed. On the control side of the delayed and immediate group, 0.182 mm2 and 0.037 mm2 osteoid extensions were observed, corresponded to the qualitative bone formation score that represented almost no new bone formation. In line with these findings, other studies have investigated bone formation surrounding unloaded dental implants and mini-screws. Jansen et al. [33] found that after six weeks, unloaded dental implants had established contact with the bone, and bone deposits and remodeling of lacunae were identified. In another study, Oh et al. [15] found that after six weeks, the bone contact of unloaded mini-screws in the mandible was greater than that of loaded mini-screws. However, this difference was not statistically significant. According to the histopathological findings of our study, inserting unloaded screws resulted in significantly greater bone formation scores than the control sides, regardless of whether the mini-screws were inserted immediately or delayed. In line with these findings, other studies have investigated bone formation surrounding unloaded dental implants and mini-screws. In order to provide proper treatment to prevent alveolar bone resorption, further investigation of the unloaded mini-screws effect on surrounding tissue will provide a deeper understanding of bone formation mechanisms.

Canine models are often utilized in dental research due to the notable similarities between human and dog bone structures more than other animal models [34]. It has been observed that both species share comparable features in terms of secondary osteons and intracortical remodeling [35]. Further similarities extend to levels of collagen and insulin-like growth factor-1 in both cortical and cancellous bones. Remarkably, research has shown that the periodontal tissue and bone mineral density of a beagle dog’s mandible align closely with human equivalents [36]. Therefore, the insights gleaned from the current study could potentially hold relevance and applicability for human individuals. In our study, only male dogs included since using a single-sex in a study increases the reproducibility of results. Moreover, selecting male dogs would be more appropriate because physiological responses and behaviors related to their reproductive cycles in female dogs increased additional variability related to hormonal fluctuations. Also, practicality, male dogs for research purposes were more available [37].

In conclusion, in this canine model, immediate mini-screw placement resulted in greater socket height preservation than delayed placement. Either immediate or delayed mini-screw insertion had no significant effect on bone width. Bone density was higher around the mini-screws than on the control side. As we proceeded from the apical point of the mini-screws entrance point to 2 mm further, the bone density difference between the experimental and control sides decreased. Compared to the immediate group, the delayed group showed better results in terms of bone density. Both groups had higher levels of bone formation on the intervention side than on the control side. The presence of inflammation on the screw side was not significantly different from the control side in either group. The extraction socket’s height, density, and histology ossification were efficiently preserved by both immediate and delayed mini-screws. The application of the current findings to the human alveolar bone and autogenous bone grafts warrants further research.

## Data Availability

The datasets used and/or analyzed during the current study available from the corresponding author on reasonable request.

## Declarations

All experimental protocols were approved by the local ethics committee of Mashhad University of Medical Sciences, Iran (IR.MUMS.DENTISTRY.REC.1400.041).
